# An Allosteric Mechanism Inferred from Molecular Dynamics Simulations on Phospholamban Pentamer in Lipid Membranes

**DOI:** 10.1371/journal.pone.0018587

**Published:** 2011-04-15

**Authors:** Peng Lian, Dong-Qing Wei, Jing-Fang Wang, Kuo-Chen Chou

**Affiliations:** 1 College of Life Science and Biotechnology and Key Laboratory of Microbial Metabolism, Shanghai Jiao Tong University, Shanghai, China; 2 Key Laboratory of Systems Biomedicine, Ministry of Education, Shanghai Center for Systems Biomedicine, Shanghai Jiao Tong University, Shanghai, China; 3 Shanghai Center for Bioinformation and Technology, Shanghai, China; 4 Gordon Life Science Institute, San Diego, California, United States of America; Aston University, United Kingdom

## Abstract

Phospholamban functions as a regulator of Ca^2+^ concentration of cardiac muscle cells by triggering the bioactivity of sarcoplasmic reticulum Ca^2+^-ATPase. In order to understand its dynamic mechanism in the environment of bilayer surroundings, we performed long time-scale molecular dynamic simulations based on the high-resolution NMR structure of phospholamban pentamer. It was observed from the molecular dynamics trajectory analyses that the conformational transitions between the “bellflower” and “pinwheel” modes were detected for phospholamban. Particularly, the two modes became quite similar to each other after phospholamban was phosphorylated at Ser16. Based on these findings, an allosteric mechanism was proposed to elucidate the dynamic process of phospholamban interacting with Ca^2+^-ATPase.

## Introduction

Phospholamban (PLN) is a 52-residue integral membrane protein that functions as a regulator of Ca^2+^ concentration of cardiac muscle cells by triggering the bioactivity of sarcoplasmic reticulum Ca^2+^-ATPase (SERCA) [1], [2]. Binding of PLN to SERCA can inhibit the SERCA Ca^2+^ pump activity so as to decrease the Ca^2+^ concentration within the cell, further leading the reduction of the relaxation rate of heart muscle cells [3], [4]. However, the inhibition will lost after PLN is phosphorylated at position 16 or 17 by cAMP- or calmodulin-dependent protein kinases, resulting in the dissolution of PLN/SERCA complex or an altered interaction between the two proteins [5], [6]. Based on a link between PLN mutations and heart failure in humans, it is found that the alteration of PLN inhibitory function can lead to degenerative cardiomyopathy [7], [8]. Thus, for its regulation of heart contractions in heart muscle cells, PLN has been of considerable interest as a potential target for the treatment of degenerative cardiac diseases.

With an aim of understanding the inhibitory mechanism of PLN, many studies have begun with the structural behaviors of PLN in the membrane or other solvents without SERCA. As reported, more than 75% of PLN in the membranes adopt the pentameric form [9], [10]. However, most experimental evidences support the fact that the inhibition of SERCA is primarily involved in the monomeric form of PLN rather than the pentameric form [11], [12], [13]. Thus, most of the theoretical and experimental studies of PLN are focused on the monomeric form of PLN [14], [15], [16], [17], [18], [19], [20]. It is noted that the monomeric PLN has three distinct structural domains: a short cytoplasmic (CP) helices (also called domain Ia or cytoplasmic domain, residues 1–16), a hinge with a β-turn type III conformation (residues 17–22), as well as a long hydrophobic transmembrane (TM) helix that is composed of domain Ib (residues 23–30) and domain II (residues 31–52) [21], [22]. Further investigations with multidimensional solid-state NMR and hybrid solution NMR give an indication that the CP and TM helices adopt angles of 93–102° and 22–24° respectively with respect to the lipid normal [17], [22]. Additionally, the pentameric PLN is reported to be able to form an ion channel for Ca^2+^ and Cl^-^ [23], [24]. Also, the pentameric form is considered to be capable of storing the monomeric form, revealing a mechanism for the cell to control inhibition of Ca^2+^-ATPase [25].

By now, the bellflower and pinwheel models are of general acceptance to be the structural models for pentameric PLN. The former is a high-resolution NMR structure determined by James J. Chou and his co-workers with PDB entry 1zll [26], [27]. In the bellflower model, PLN pentamer shows a pore-forming coiled-coil structure with the TM helices remarkably bending away from the channel pore near the cytoplasmic side. The latter is a theoretical model (PDB entry 1xnu) obtained from the Forster resonance energy transfer measurements [28]. In this model, TM helices are less bended compared with the bellflower model, and the cytoplasmic helices are located in the plane of the membranes.

Although several MD studies of partial or full length PLN in different environments have been reported [15], [18], [19], [20], [29], [30], [31], most of these studies are only focused on the monomeric PLN. So far only two papers [30], [31] are involved with the pentameric PLN. However, the one published by Kim et al. [30] only used the bellflower models, while the other [31] did not consider the phosphorylated PLN at all. In view of this, the present study was initiated to use all-atom molecular dynamics (MD) simulations to study the conformational dynamics of both bellflower and pinwheel models of PLN pentamer as well as their phosphorylated forms in POPC bilayer surroundings, so as to gain the atomistic insights into the internal motion [32], [33] of PLN pentamer and its functions. Similar approaches have been successfully used to investigate various problems related to protein structure [34], [35], [36], [37], [38], [39], protein-protein interactions and protein-drug interactions [40], [41], [42], [43], as well as drug design [44], [45], [46], [47].

## Materials and Methods

The initial coordinates for the bellflower and pinwheel models of PLN pentamer were taken from the RCSB Protein Data Bank [48] with PDB entries 1zll [26] and 1xnu [28], respectively. Based on the atomic coordinates of the bellflower structure (1zll), the following four initial systems were generated for MD simulation studies: (i) monomeric PLN as denoted by PLN^bf-mono^; (ii) pentameric PLN denoted by PLN^bf-penta^; (iii) phosphorylated PLN monomer denoted by _p_PLN^bf-mono^; (iv) phosphorylated PLN pentamer denoted by _p_PLN^bf-penta^. Likewise, based on the atomic coordinates of the pinwheel structure (1xnu), the following four initial systems were generated: (i) monomeric PLN as denoted by PLN^pw-mono^; (ii) pentameric PLN denoted by PLN^pw-penta^; (iii) phosphorylated PLN monomer denoted by _p_PLN^pw-mono^; (iv) phosphorylated PLN pentamer denoted by _p_PLN^pw-penta^.

Before performing MD simulations, the aforementioned eight systems were merged with a patch of a pre-equilibrated membrane with 256 POPC bilayers, and subsequently solvated in TIP3P water molecules [49]. Meanwhile, sodium ions were randomly placed to neutralize the simulated systems. Subsequently, 150,000-step energy minimization was performed on water molecules and ions for the monomeric systems using conjugate gradient and line search algorithm to remove energetically unfavorable contacts. Another 50,000 steps were added for the pentameric systems because of their larger sizes. In this procedure, both of the proteins and lipids were fixed. Then, all the systems were heated to 310 K over 500 ps by velocity rescaling, and equilibrated for 4 ns in the NPT ensemble with all non-hydrogen atoms of the protein and POPC membranes harmonically restrained. The equilibration was continued for another 10 ns with the harmonic restrain applied to the protein backbone only. Finally, the systems obtained at the end of the aforementioned equilibrations were used for the further unrestrained 40-ns MD simulations. As the simulated systems may be far too large to expect the statistical convergence in 40-ns MD simulations, we have done some repeats with different starting structures and starting vectors. Thus, for each simulation, at least 10 MD trajectories with different starting vectors were generated.

All the MD simulations involved in this study were performed by NADM 2.6 package [50] with the CHARMM27 force field parameters [51], periodic boundary conditions, and NPT ensemble. SHAKE algorithm with a tolerance of 10^−6^ Å was used to constrain all bonds involved with hydrogen atoms. The Nosé-Hoover Langevin piston pressure control and Langevin damping dynamics were employed to keep the simulated systems at a constant pressure of 1 atmosphere and a constant temperature of 310 K [52], [53]. The long-range electrostatic interactions were evaluated by particle mesh Ewald (PME) method [54] with the size of the grid at about 1Å. The van der Waals interactions were treated by using a cutoff of 12Å. All the simulations were performed with a time step of 1fs, and the coordinates were saved every 1ps.

## Results and Discussion

### Global properties

As a crucial criterion for the convergence measure of the protein systems concerned, the root mean square (RMS) deviation from the initial structures of the C^α^ atoms for both bellflower and pinwheel models of pentameric and monomeric PLN and _p_PLN systems were calculated. Both bellflower and pinwheel models of pentameric PLN and _p_PLN systems underwent various conformational changes during our 40-ns MD simulations. For the bellflower models, the overall RMS deviation value for PLN was around 7.52±0.68 Å, while that for _p_PLN was 7.50±1.21 Å. For the pinwheel model, the overall RMS deviation value for PLN was 8.29±1.45 Å, while that for _p_PLN was 9.67±1.61 Å. There was a difference of about 1 Å for the overall RMS deviations between the two models in PLN, indicating that the pinwheel model was more flexible, and involved with larger conformational changes. It is interesting to note that the phosphorylation at Ser16 remarkably enhanced this kind of difference to around 1.4 Å. In view of this, it is very likely that the phosphorylation could play a role in increasing the flexibility of both structural models.

**Figure 1:**
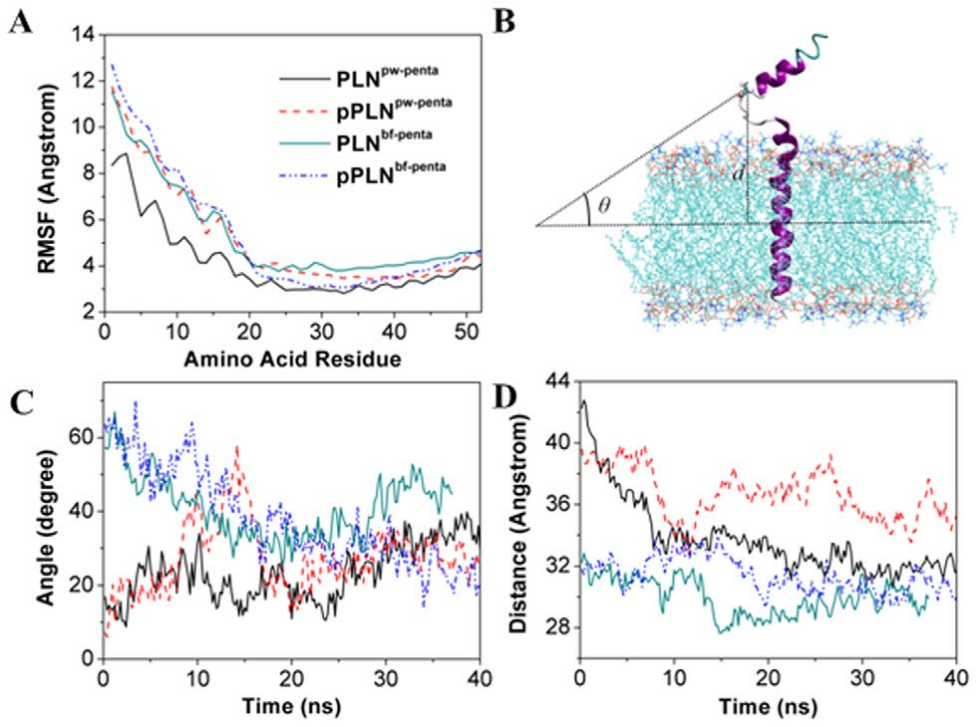
Global properties for the MD simulations. (A) Time dependent RMS fluctuations of all the C^α^ atoms, (B) Definitions of the angle *θ* and distance *d*, (C) Time dependent angle *θ* value, and (D) Time dependent distance *d* value. The black, green, red, and blue curves stand for pinwheel PLN, bellflower PLN, pinwheel _p_PLN, and bellflower _p_PLN, respectively.

However, such RMS differences are really small for a biomacromolecular system. One possible explanation for this observation is that most residues of PLN have little conformational changes. As aforementioned, PLN monomer has three distinct structural domains: a short cytoplasmic domain (domain Ia), a hinge with a β-turn type III conformation, and a long hydrophobic transmembrane domain (domain Ib and II). The long transmembrane domain, which occupies almost 60% residues of PLN monomer, is inserted into the lipid bilayer with little room for large amplitude fluctuations. Therefore, the transmembrane domain has little contributions to the RMS differences. In other words, the major contributions to the RMS differences were from the cytoplasmic and the β-turn, which contain much less residues than the transmembrane domain. To further confirm this, we calculated the RMS fluctuation values for each residue in both bellflower and pinwheel models of pentameric PLN and _p_PLN systems. The results are shown in Fig. 1A, from which we can see that for both the bellflower and pinwheel models, the RMS fluctuation values in the transmembrane regions were almost the same. However, the diversity was detected at the position of res.10–20 that was situated outside of the bilayers, belonging to the cytoplasmic domain where the phosphorylation occurred.

### Structure and dynamics of the cytoplasmic domain

In order to study the structural and dynamic diversity of the cytoplasmic domain, in Fig. 1B we defined an angle *θ* to describe the direction of the cytoplasmic domain to the POPC bilayers, and a distance *d* to describe the distance of the cytoplasmic domain with the POPC bilayers. Based on the 10 independent simulations for each simulated system (40-ns MD trajectories), time averages were used to compute expectation values for the angle *θ* and distance *d*. Following similar analyses by some theoretical studies [55], [56], bootstrap-style sampling-with-replacement approach was adopted to generate new re-sampled datasets for each simulation trajectory, each of which was used to estimate average values for the angle *θ* and distance *d*. The distribution of these average values over all re-sampled dataset for all the MD trajectories was subsequently employed to estimate the variability of the angle *θ* and distance *d*, and also to calculate variances and confidence intervals.

The angle *θ* was considered as the angle of the cytoplasmic domain with the lipid bilayer. The first and last residues in the cytoplasmic domain were used to determine a line, which was further used to determine the angle *θ*. With the development of simulation along time, the *θ* value for the bellflower model was about 41.19±8.46 degrees, while that for the pinwheel model was about 23.40±7.46 degrees (Fig. 1C). The differences in the *θ* value indicated that bellflower and pinwheel structures assumed quite different conformations in the cytoplasmic domain: the former was extended to the region away from the bilayers, while the latter bended to near the bilayers. However, after phosphorylation at Ser16, both the two models shared a similar *θ* value (about 30 degrees,), indicating that the two were with quite the same configurations due to the phosphorylation effect.

It can be seen from Fig. 1D that the *d* value for the bellflower model was about 30.00±1.19 Å, while that for the pinwheel model was about 33.72±2.40 Å. After phosphorylation at Ser16, the distance *d* for both models increased: for the bellflower structure, it was increased from 30 Å to 32 Å; for the pinwheel mode, it was increased from 32 Å to 37 Å. These evidences further indicate that phosphorylation at Ser16 could enhance the flexibility of PLN as mentioned above. This finding is also quite consistent with the NMR study in DPC micelles [14] and previous MD simulations [18], [19], [20], [30].

### Structural transition in the cytoplasmic domain

**Figure 2:**
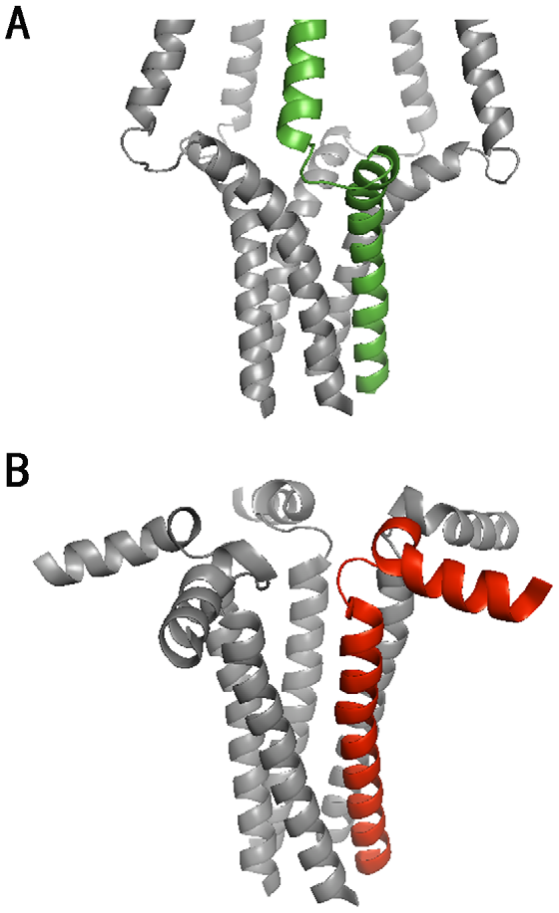
The crystal structures of the bellflower and pinwheel models for the pentameric PLN. (A) In the bellflower model, PLN assumes “R”-state configuration, and (B) in the pinwheel model, PLN assumes “L”-state configuration.

**Figure 3:**
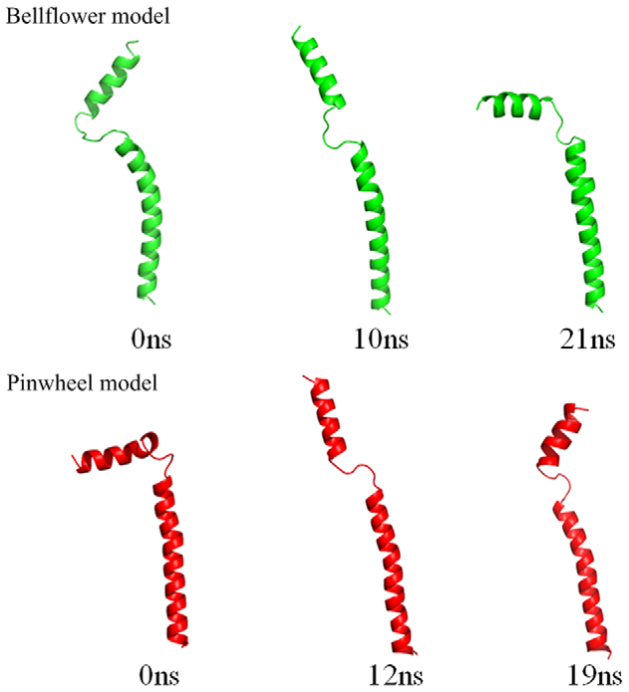
Structural transitions between the “R” and “L” states. In this snapshot figure, only one subunit of the pentamer is shown for the bellflower model (green) and pinwheel model (red), respectively.

The structural differences in the bellflower and pinwheel models are mainly located in the cytoplasmic domain. In general, the bellflower structure assumes an extended appearance; while the pinwheel model assumes a bent conformation having more interactions with the bilayers (Fig. 2). The cytoplasmic helices in the bellflower assembly are oriented at ∼20 degrees with respect to the bilayers, while the ones in the pinwheel assembly are ∼90 degrees. It is intriguing to have observed the structural transitions between the two models via our MD simulations. As shown in Fig. 3, for the bellflower model, although the initial structure was in the “extended” state (*θ* value is 57.43 degrees, and *d* value is 32.23 Å), after 21-ns simulations it became the “bent” state (*θ* value is 24.95 degrees, and *d* value is 31.86 Å) as shown in the pinwheel model. Likewise, for the pinwheel model, similar transition was detected after 19-ns simulations when it changed its original “bent” conformation (*θ* value is 16.91 degrees, and *d* value is 42.28 Å) to the “extended” one (*θ* value is 39.34 degrees, and *d* value is 30.57 Å). These findings were also supported by the experimental results that both extended and bent states of PLN have been readily detected in the micelles and lipid bilayers using electron paramagnetic resonance (EPR) spectroscopy [57], [58]. Further analyses of chemical shift perturbation for the PLN-SERCA complex showed clear evidences for the structural transition between the extended and bent states [57], [59].

In the _p_PLN systems, the conformations from both the bellflower structure and pinwheel structure look almost the same. As shown in Fig.1C, after 20-ns MD simulations the angle *θ* values for both _p_PLN systems are 30.87±9.93 degrees (bellflower model) and 27.37±9.05 degrees (pinwheel model). One possible explanation for this observation is that phosphorylation can increase the interactions of PLN with the lipid bilayer, resulting in the fact that the cytoplasmic domain no longer moves as freely as the unphosphorylated structure. Thus, after phosphorylation at Ser16, both models have to adopt a similar configuration, which is also supported by the previous theoretical studies [19], [20].

**Figure 4:**
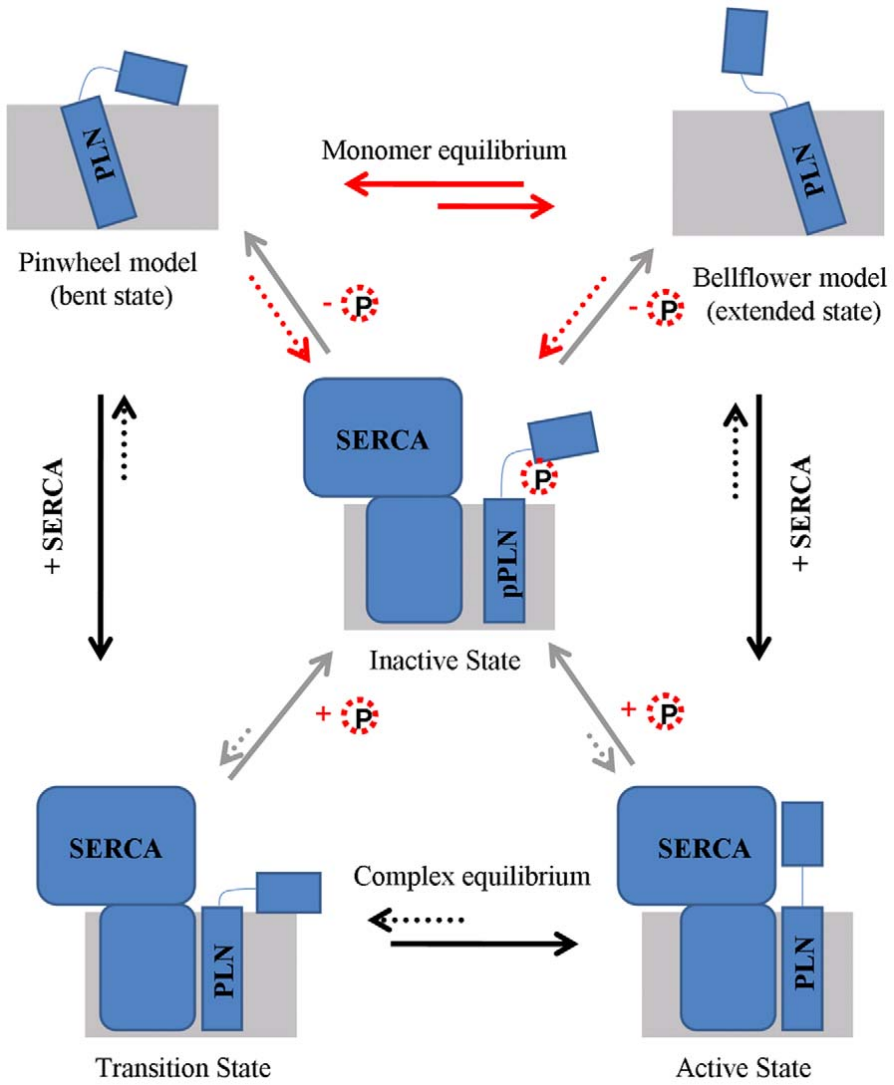
A proposed allosteric model for the regulation mechanism of SERCA by PLN. In this figure, the membrane and PLN are represented by rectangles and colored in gray and blue, respectively. SERCA is shown in blue boxes. The arrows in black show the formation of SERCA-PLN complex. The gray arrows are for the phosphorylation and dephosphorylation. Reactions shown with red arrows are the processes we found in this study. Phosphoric group is represented by the red dotted circle.

Accordingly, our theoretical studies supported the allosteric model to elucidate the interactions between PLN and SERCA, as illustrated in Fig. 4. According to the allosteric model, there is a dynamic equilibrium between the “extended” and “bent” conformations for PLN in the free form. The major difference between the extended and bent states for PLN is located on the cytoplasmic domain. For the extended state of PLN (bellflower model), the cytoplasmic domain is in a dynamically disordered state in which the central part of this domain is unfolded. For the bent state of PLN (pinwheel model), this domain employs an ordered state. Both models can interact with SERCA, forming transition and active states respectively. Only the bellflower structure with the “extended” conformation can form a thermodynamically more stable complex (active state) with SERCA so as to inhibit the activity of Ca^2+^ pump. This is because the bent state of PLN is not propitious for the SERCA binding by the reason that the cytoplasmic domain in this state is much close to the lipid, far away from SERCA. Owing to the disordered structure, the extended state of PLN can be extended above the lipid surface and interact with SERCA.

However the phosphorylation at Ser16 reduces the structural difference between the PLN monomer in bellflower model and that in pinwheel model, revealing a structural transition between the ordered and disordered states in the cytoplasmic domain. This order-to-disorder or disorder-to-order transition can influence the conformational equilibrium between the bent and extended states of PLN monomers. Specifically, phosphorylation can shift the conformational equilibrium towards the extended state via a cooperative effect [60]. Furthermore, the phosphorylation at Ser16 can also inactive the SERCA-PLN complex, independent of it bding in either the transition state or in the active state, by disrupting the interactions between SERCA and PLN. According to the experimental and theoretical studies, a crucial hydrogen bond formed by PLN (Gln26) and SERCA (Arg234) maybe lost. In this case, the activity of Ca^2+^ pump is recovered. Interestingly, the dephosphorylation of Ser16 may drive PLN to the original equilibrium again so as to make the regulation cycle move on. Using the allosteric model, our findings show for the first time how the two different conformations of PLN monomer are produced. The results of this study also reveal the mechanism of SERCA Ca^2+^ pump's regulation by PLN.
